# Workflows in bioinformatics: meta-analysis and prototype implementation of a workflow generator

**DOI:** 10.1186/1471-2105-6-87

**Published:** 2005-04-07

**Authors:** Alexander Garcia Castro, Samuel Thoraval, Leyla J Garcia, Mark A Ragan

**Affiliations:** 1 Institute for Molecular Bioscience, The University of Queensland, Brisbane, Qld 4072, Australia; 2 Australian Research Council (ARC) Centre in Bioinformatics, Australia; 3 LIBROPHYT, Bioinformatique, Centre de Cadarache, Bâtiment 185, DEVM, 13108 St Paul-Lez-Durance, France; 4 Universidad de la Sabana, Bogota, Colombia

## Abstract

**Background:**

Computational methods for problem solving need to interleave *information access *and *algorithm execution *in a problem-specific workflow. The structures of these workflows are defined by a scaffold of syntactic, semantic and algebraic objects capable of representing them. Despite the proliferation of GUIs (Graphic User Interfaces) in bioinformatics, only some of them provide workflow capabilities; surprisingly, no meta-analysis of workflow operators and components in bioinformatics has been reported.

**Results:**

We present a set of syntactic components and algebraic operators capable of representing analytical workflows in bioinformatics. Iteration, recursion, the use of conditional statements, and management of suspend/resume tasks have traditionally been implemented on an *ad hoc *basis and hard-coded; by having these operators properly defined it is possible to use and parameterize them as generic re-usable components. To illustrate how these operations can be orchestrated, we present GPIPE, a prototype graphic pipeline generator for PISE that allows the definition of a pipeline, parameterization of its component methods, and storage of metadata in XML formats. This implementation goes beyond the macro capacities currently in PISE. As the entire analysis protocol is defined in XML, a complete bioinformatic experiment (linked sets of methods, parameters and results) can be reproduced or shared among users. *Availability: * (interactive),  (download).

**Conclusion:**

From our meta-analysis we have identified syntactic structures and algebraic operators common to many workflows in bioinformatics. The workflow components and algebraic operators can be assimilated into re-usable software components. GPIPE, a prototype implementation of this framework, provides a GUI builder to facilitate the generation of workflows and integration of heterogeneous analytical tools.

## Background

Computational approaches to problem solving need to interleave *information access *and *algorithm execution *in a problem-specific workflow. In complex domains like molecular biosciences, workflows usually involve iterative steps of querying, analysis and optimization. Bioinformatic experiments are often workflows; they link analytical methods that typically accept an input file, compute a result, and present an output file. Most tool-driven integration approaches have so far addressed the problem of providing a single GUI for a set of analytical methods. Combining methods into a flexible framework is usually not considered. Analytical workflows provide a path to discover information beyond the capacities of simple query statements, but are much less easy to implement within a common environment.

Workflow management systems (WFMS) are basically systems that control the sequence of activities in a given process [1]. In molecular bioscience, these activities can be divided among those that address query formulation, and those that focus more on analysis. At this abstract level, WFMS could serve to control the execution of both query and analytical procedures. All of these procedures involve the execution of activities, some of them manual, some automatic. Dependency relationships among them can be complex, making the synchronization of their execution a difficult problem.

One dimension of the complexity of workflows in molecular biosciences is given by the various transformations performed on the data. Syntactic (operational) interoperability establishes the possibility for data to be piped from one method into another. Semantic issues (another dimension) arise from the fact that we need to separate domain knowledge from operational knowledge. We should be able to describe a task of configuring a workflow from its primary components according to a required specification, and implement a program that realizes this configuration independently of the workflow and components themselves.

Biologists provide rich descriptions of their experiments (materials and methods) so they can be easily replicated. Once techniques have been standardized, usually this knowledge is encapsulated in the form of an analytical protocol. With *in silico *experiments as well, analytical protocols make it possible for experiments to be replicated and shared, and (*via *meta-information) for the knowledge behind these workflows to be captured. These protocols should be reproducible, ontology-driven, curated internally, and annotated externally.

Systems such as W2H/W3H [2] and PISE [3] provide some tools that allow methods to be combined. W3H is a task framework that allows the methods available under W2H [4] to be integrated; however, those tasks have to be hard-coded. In the case of PISE, the user can either define a macro using Bioperl , or use the interface provided and register the resulting macro. In either case, it is assumed that the user can program, or script in Perl. Macros cannot be exchanged between PISE and W2H, although these two systems provide GUIs for more or less the same set of methods (EMBOSS: [5]). Indeed, macros cannot be easily shared even among PISE users. Biopipe , on the other hand, provides integration for some analytical tools using Bioperl API (Application Programming Interface) using MySQL to store results as well as the workflow definition; in this way, users are able to store results in MySQL and monitor the execution of the pre-defined workflow.

The TAVERNA  project provides similar capabilities to those offered by GPIPE. However, on one hand inclusion of new analytical methods is not currently possible since no GUI generator is provided, and on the other hand as TAVERNA is part of myGrid [6] it follows a different integrative approach (Web Services). Pegasys [7] is a similar approach, going beyond analytical requirements and providing database capacities.

GPIPE provides a real capacity for users to define and share complete analytical workflows (methods, parameters, and meta-information), substantially mitigating the syntactic complexity that this process involves. Our approach addresses overall collaborative issues as well as the physical integration of tools. GPIPE provides an implementation that builds on a flexible syntactic structure and a set of algebraic operations for analytical workflows. For testing purposes we provide a simple example of a workflow (inference of a phylogeny of rodents) that involves piping among three methods. Although here their execution takes place on a common server, it is equally possible to distribute the process over a grid using GPIPE.

## Results

Our workflow follows a task-flow model; in bioinformatics, tasks can be understood as analytical methods. If workflow models are represented as a directed acyclic graph (DAG), analytical methods then appear as nodes, and state information is represented as conditions attached to the edges. Our syntactic structure and algebraic operators can be used to represent a large number of analytical workflows in bioinformatics; surprisingly, there are no other algebraic operators reported in the literature capable of symbolizing the different operations required for analytical workflows in bioinformatics (or, indeed, more broadly in e-science, although they are widely used in the analysis of business processes). Different groups have developed a great diversity of GUIs for EMBOSS and GCG, but a meta-analysis of the processes within which these analytical implementations are immersed is not yet fully available. Some of the existing GUIs have been developed to make use of grammatical descriptions of the analytical methods, but there exists no standard meta-data framework for GUI and workflow representation in bioinformatics.

### Syntactic and algebraic components

**Figure 1:**
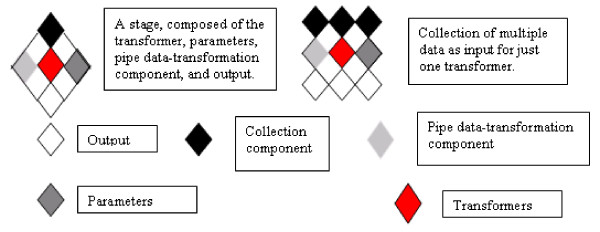
Syntactic components describing bioinformatic analysis workflows.

Our workflow conceptualization (Figure 1) closely follows those of Lei and Singh [8] and Stevens *et al. *[9]. We have adapted these meta-models to processes in bioinformatic analysis. We consider an *input/output data object *as a collection of input/output data. For us a *transformer *is the atomic work item in a workflow. In analytical workflows, it is an implementation of an analytical algorithm (analytical method). A *pipe component *is the entity that contains the required input-output relation (*e.g. *information about the previous and subsequent tasks); it assures syntactic coherence. Our workflow representation has *tasks*, *stages*, and *experimental conditions (parameters)*. In our view, *protocols *are sets of information that describe an experiment. A protocol contains workflows, annotations, and information about the raw data; therefore we understand a *workflow *to be a group of stages with interdependencies. It is a process bound to a particular resource that fulfils the analytical necessities.

We identify needs common to analytical workflows in bioinformatics:

• Flexibility in structuring and modelling (open-ended, sometimes *ad hoc *workflow definition, allowing decision-making whilst a workflow is being executed).

• Support for workflows with a complex (or nested) inner structure of individual steps (such that multi-level modelling becomes appropriate). Biological workflows may be complex not simply because of the discrete number of steps, but due to the highly nested structure of iteration, recursion and conditional statements that, moreover, may involve interaction with non-workflow systems.

• Distribution of workflow execution over grid environments.

• Management of failures. This particular requirement is related to conditional statements: *where *the service will be executed should be evaluated based on considerations of availability and efficiency made previous to the execution of the workflow. In situations where a failure halts the process, the system should either recover it, or dispatch it somewhere else without requiring intervention by the user.

• System functionality features such as browsing and visualization, documentation, or coupling with external tools, *e.g. *for analysis.

• A semantic layer for collaborative purposes. This semantic layer has many other features, and may be the foundation for intelligent agents that facilitate collaborative research.

Executing these bioinformatic workflows further requires:

• Support for long-running activities with or without user interaction.

• Application-dependent correctness criteria for execution of individual and concurrent workflows.

• Integration with other systems (*e.g*. file managers, database management systems, product data managers) that have their own execution/correctness requirements.

• Reliability and recoverability with respect to data.

• Reliable communication between workflow components and processing entities.

**Figure 2:**
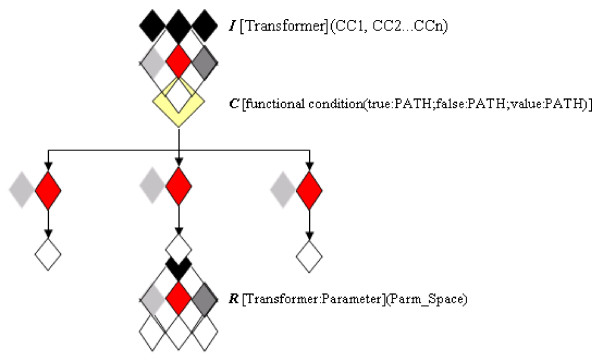
Syntactic components and algebraic operators.

Among these types of requirements, we focus our analysis only on those closely related to workflow design issues, more specifically (a) the piping of data, (b) the availability of conditional statements, (c) the need to iterate one method over a set of different inputs, (d) the possibility of recursion over a parameter space for a method, (e) and the need for stop/break management. Algebraic operators can accurately capture the meaning of these functional requirements. To describe an analytical workflow, it is necessary to consider both algebraic operators and syntactic components. In Table 1 we present the definition of those algebraic operators we propose, and in Figure 2 we illustrate how these operators and syntactic elements together can describe an analytical workflow.

*Iteration *is the operator that enables processes in which one transformer is applied over a multiple set of inputs. A special case for this operator occurs when it is applied over a blank transformer; this case results in replicates of the input collection. Consider an analytical method, or a workflow, in which the same input is to be used several times; the first step would be to use as many replicates of the input collection as needed. The *recursion *operation takes place when one transformer is applied with parameters defined not as a single value, but as a range or as a set of values. The *conditional* operator has to do with the conditioned execution of transformers. This operation can be attached to a function evaluated over the application of a recursion, or of an iteration; if the stated condition is true, then the workflow executes a certain path. *Conditional statements *may also be applied to cases where an argument is evaluated on the input; the result affects not a path, but the parameter space of the next stage. The *suspension/resumption *operation stands for the capacity of the workflow to stop and re-capture the jobs.

Formal Concept analysis (FCA) is a mathematical theory based on ordered sets and complete lattices. Numerous investigations have shown the usefulness of concept lattices for information retrieval combining query and navigation, learning and data-mining, visual constructors and visual programming [10]. FCA helps one to define valid objects, and identify behaviours for them. We are currently working on a complete FCA for biological data types and operations (database and analytical). Here we define operators in terms of pre- and post-conditions, as a step toward eventual logical formalization. We focus on those components of the discovery process not directly related to database operations; a good integration system will "hide" the underlying heterogeneity, so that one can query using a simple language (which views all data as if they are already in the same memory space). Selection of the query language depends only on the data model. For the XML "data model", XML-QL, XQL, and other XML query languages are available. For the nested relational model there are nested relational calculi and nested relational algebras. For the relational model SQL, relational algebras and so on are available. For database operations, the issues that arise are lower-level (*e.g. *expression of disk layout, latency cost, etc. in the context of query optimisation), and it is not clear that any particular algebra offers a significant advantage.

**Operator**: **Iteration **(**I**): **I**[Transformer, (*CC1, CC2, ..., CCn*)]: (CC1', CC2', ..., CCn')

**Pre-condition**:

T = Transformer ∧ T ≠ ***blank***

C = {*CC1, CC2, ..., CCn*} such that *CCi *∈ { Biological data types }

**Post-condition**:

C' = {CC'1, CC'2, ..., CC'n} such that CC'i = T(*CCi*) ∧ 1 ≤ i ≤ n

**Operator**: **Iteration **(**I**): **I**[***blank***: *num*, (*CC1, CC2, ..., CCn*)]: (CC1, CC2, ..., CCn)1, (CC1, CC2, ..., CCn)2, ..., (CC1, CC2, ..., CCn)*num*

**Pre-condition**:

*num *∈ , num = number of replicates.

C = {*CC1*, *CC2*, ..., *CCn*} such that *CCi *∈ { Biological data types }

**Post-condition**:

C' = {CC'1, CC'2, ..., CC'n} such that CC'i = *CCi *∧ 1 ≤ i ≤ n

**Operator**: **Recursion **(**R**): **R**[Transformer: *Parameter*, (*Parm_Space*)]: Parm_Space'

**Pre-condition**:

P = *Parameter *such that P ∈ *Parm_Space *∨ (*Parm_Space *= { *Parm_Values *} ⋁ *Parm_Space *= { *Parm_Values *})

T = Transformer

**Post-condition**:

Parm_Space' = T (*Parm_Space*)

**Operator**: **Condition **(**C**): **C**[Functional_Condition]: PATH

**Pre-condition**:

FC = Functional_Condition

**Post-condition**:

PATH = true ∨ false ∨ value

**Operator**: **Suspension/Resumption **(**S**): **S**[*re-take, jobs*]: Execution

**Pre-condition**:

(*Re-take *= true) ∨ (*Re-take *= false ∧ *jobs *= Set of jobs which should be suspended)

**Post-condition**:

(*Re-take *= true ∧ ((Execution = true ∧ Previously suspended jobs are re-taken)∨ (Execution = false ∧ There were no suspended jobs))) ∨ (*Re-take *= false ∧ (Execution = true ∧ ∀ j such that j ∈ *jobs*, j is suspended))

**Figure 3:**
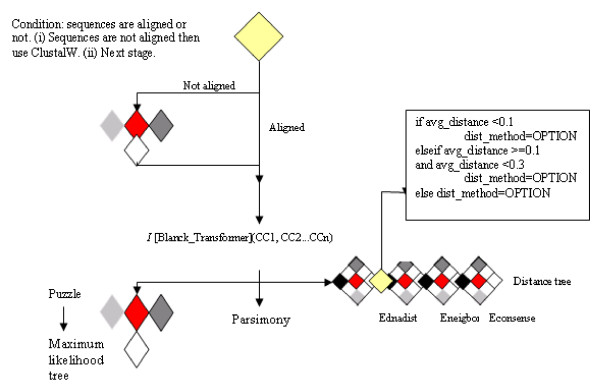
Phylogenetic analysis workflow.

A more-detailed example involves the inference of molecular phylogenetic trees by executing software that implements three main phylogenetic inference methods: distance, parsimony and maximum likelihood. Figure 3 illustrates how our algebraic operators and syntactic components define the structure of this workflow.

**Figure 4:**
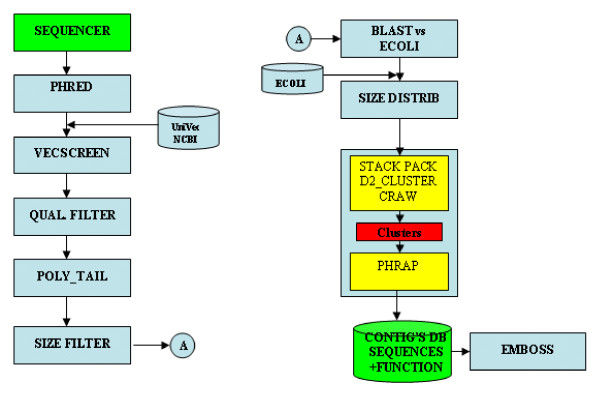
Case workflow.

In collaboration with CIAT (Center for International Tropical Agriculture, Cali, Colombia) we have implemented an annotation workflow using standard technology (GPIPE/PISE) and web services (TAVERNA). Our case workflow is detailed in Figure 4.

Implementation of both of these workflows was a manual process. GUI generation was facilitated by using PISE as our GUI generator, and this simplified the inclusion of new analytical methods as needed. Database calls had to be manually coded in both cases. Choreographing the execution of the workflow was not simple, as neither has a real workflow engine. It proved easier to give users the ability to manipulate parameters and data with PISE/GPIPE, partly due to wider range of methods within BioPerl partly because algebraic operators were readily available as part of PISE/GPIPE. From this experience we have concluded that, due to the immaturity of current available web service engines, it is still most practical to implement simple XML workflows that allow users to manipulate parameters, use conditional operators, and carry out write and read operations over databases. This balance will, of course, presumably shift as web services mature in the bioinformatics applications domain.

### Workflow generation, an implementation

We have developed GPIPE, a flexible workflow generator for PISE. GPIPE extends the capabilities of PISE to allow the creation and sharing of customised, reusable and shareable analytical workflows. So far we have implemented and tested GPIPE over only the EMBOSS package, although extension to other algorithmic implementations is possible where there is an XML file describing the command-line user interface.

Workflows automate businesses procedures in which information or tasks are passed between conforming entities according to a defined set of rules; some of these business rules are defined by the user, and in our implementation are managed *via *GPIPE. For our purposes, the conforming entities are analytical methods (Clustal, Protpars, etc.). Syntactic rules drive the interaction between these entities (*e.g. *to ensure syntactic coherence between heterogeneous file formats). GPIPE also assures the execution of the workflow, and makes it possible to distribute different jobs over a grid of servers. GPIPE addresses these requirements using mostly Bioperl.

In GPIPE, each analysis protocol (including any annotations, *i.e. *meta-data) is defined within an XML file. A Java applet provides the user with an exploratory tool for browsing and displaying methods and protocols. Synchronization is maintained between client-side display and server-side storage using Javascript. Server-side persistence is maintained through serialized Perl objects that manage the workflow execution. GPIPE supports independent branched tasks in parallel, and reports errors and results into an HTML file. The user selects the methods, sets parameters, defines the chaining of different methods, and selects the server(s) on which these will be executed. GPIPE creates an XML file and a Perl script, each of which describes the experiment. The Perl file may later be used on a command-line basis, and customized to address specific needs. The user can monitor the status of workflow execution, and access intermediary results. A workflow built with GPIPE can distribute its analyses onto different, geographically disperse GPIPE/PISE servers.

## Discussion

The syntactic and algebraic components we introduce above make it possible to describe analytical workflows in bioinformatics precisely yet flexibly. Detailed algebraic representation for these kinds of processes have not previously been used in this domain, although they are commonly used to represent business processes. Since open projects such as Bioperl or Biopipe contain the rules and logic for bioinformatic tasks, we believe that having an algebraic representation could contribute importantly to the development of a biological "*language*" that allows developers to avoid the tedious parsing of data and analytical methods so common in bioinformatics. The schematic representation for workflows in bioinformatics that we present here could evolve to cover other tool-driven integrative approaches such as those based on web services. Workflows in which concrete executions take place over a grid of web services involve basically the same syntactic structure and algebraic operators; however, a clear business logic needs to be defined beforehand for those web services in order to deepen the integration beyond simply the fact of remote execution. A higher level of sophistication for the pipe component as well as for the conditional operator may be needed, since remote execution requires (for example) assessment and availability of the service for the job to be successfully dispatched and processed. For our implementation we use two agents, one on the client side and the other on the server side, with the queue handled by PBS (Portable Batch System). It is possible to add a semantic layer, thereby allowing conceptual selection of the transformers; clear separation between the operational domain and the knowledge domain be would then be achieved naturally.

Semantic issues are particularly important with these kinds of workflows. An example may be derived from Figure 3, where three different phylogenetic analysis workflows are executed. These may be grouped as equivalent, but are syntactically different. Selection should be left in the hands of the user, but the system should at least inform about this similarity.

Despite agreement on the importance of semantic layers for integrative systems, such a level of sophistication is far from being achieved. Lack of awareness of the practical applications of such technologies is well illustrated with a traditional and well-studied product: Microsoft Word^®^. With Word, syntactic verification can take place as the user composes text, but no semantic corroboration is done. For two words like "purpose" and "propose", Word advises on syntactic issues, but gives no guidance concerning the context of the words. Semantic issues in bioinformatic workflows are more complex, and it is not clear if existing technologies can effectively overcome these problems.

Transformers and grid components are intrinsically related because the services are *de facto *linked to a grid component. It has been demonstrated that the use of ontologies facilitates interoperability and the deployment of software agents [11]; correspondingly, we envision semantic technology supporting the agents to form the foundation of future workflow systems in bioinformatics. The semantic layer should make the agents more aware of the information.

More and more GUIs are available in bioinformatics; this can be seen in the number of GUIs for EMBOSS and GCG alone. Some of them incorporate a degree of workflow capability, more typically a simple chaining of analytical methods rather than flexible workflow operations. A unified metadata model for GUI generation is lacking in the bioinformatics domain. Web services are relatively easy to implement, and are becoming increasingly available as GUI systems are published as web services. However, web services were initially developed to support processes for which the business logic is widely agreed upon, well-defined and properly structured, and the extension of this paradigm to bioinformatics may not be straightforward.

Automatic service discovery is an intrinsic feature of web services. The accuracy of the discovery process necessarily depends on the ontology supporting this service. Systems such as BioMoby and TAVERNA make extensive use of service discovery; however, due to the difficulty in describing biological data types, service discovery is not yet accurate. It is not yet clear whether languages such as OWL can be developed to describe relations between biological concepts with the required accuracy. Integrating information is as much a syntactic as a semantic problem, and in bioinformatics these boundaries are particularly ill-defined.

Semantic and syntactic problems were also identified from the case workflow described in Figure 4. There, we saw that to support the extraction of meaningful information and its presentation to the user, formats should be ontology-based and machine-readable, *e.g. *in XML format. Lack of these functional features makes manipulation of the output a difficult task that is usually addressed by use of parsers specific to each individual case. For workflow development, human readability can be just as important. Consider, for example, a ClustalW output where valid elements could be identified by the machine and presented to the user together with contextual menus including different options over the different data types. In this way the user would be able to decide what to do next, where to split a workflow, and over which part of the output to continue or extend the analysis. Inclusion of this functionality would allow the workflow to become more concretely defined as it is used.

Failure management is an area in which we can see a clear difference between the business world and bioinformatics. In the former, processes rarely take longer than an hour and are not so computationally intensive, whereas in bioinformatics, processes tend to be computationally intensive and may take weeks or months to complete. How failures can be managed to minimize losses will clearly differ between the two domains. Due to the immaturity of both web services and workflows in bioinformatics, it is still in most cases more practical to hard-code analytical processes. Improved failure management is one of the domain-specific challenges that faces the application of workflows in bioinformatics.

So far we have intentionally referred to GUIs and workflows as more-or-less independent. A glimpse into the corresponding metadata reveals that GUIs are themselves components of workflow systems. In the bioinformatics domain this relationship is particularly attractive, since algebraic operations are usually highly nested. The interface system should therefore provide a programming environment for non-programmers. The language as such is not complex, but makes extensive use of statements such as *while...do*, *if...then...else*, and *for...each*. The representation should be natural to the researcher, separating the knowledge domain from the operational domain.

## Conclusion

We have developed GPIPE, a flexible workflow generator that makes it possible to export workflow definitions either as XML or Perl files (which can later be handled *via *the Bioperl API). Our XML workflow representation is reusable, execution and edition of those generated workflows is possible either via the BioPerl API or the provided GUI. Each analysis is configurable, as users are presented with options to manipulate all available parameters supported by the underlying algorithms. Integration of new algorithms, and Grid execution of workflows, are also possible. Most available integrative environments rely on parsers or syntactic objects, making it difficult to integrate new analytical methods into workflow systems. We are planning to develop a more wide-ranging algebra that includes query operations over biological databases as well as different ontological layers that facilitate data interoperability and integration of information where possible for the user. We do not envision GPIPE to be a complete virtual laboratory environment; future releases will provide a content management system for bioinformatics with workbench capacities developed on top of ZOPE . We have tested our implementation over SUSE and Debian Linux, and over Solaris 8.

## Authors' contributions

AGC was responsible for design and conceptualization, took part in implementation, and wrote a first draft of the manuscript. ST was the main developer of GPIPE. LJG assisted with server issues and FCA. MAR supervised the project and participated in writing the manuscript.

## Figures and Tables

**Figure 5:**
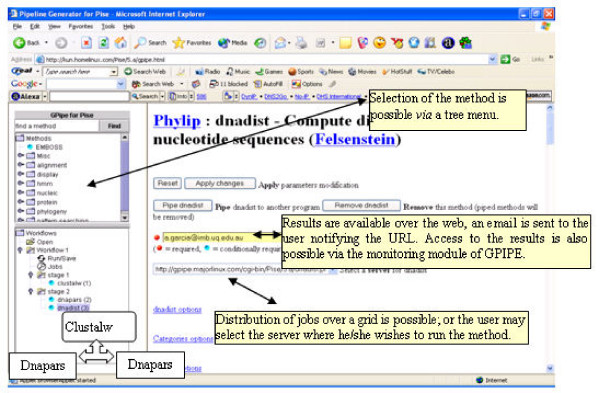
GPIPE.

**Table 1:** Algebraic operators

Operator: Iteration (***I***)	***I ***[Transformer](CC1, CC2...CCn)
Operator: Recursion (***R***)	***R ***[Transformer: Parameter](Parm_Space)
Operator: Condition (***C***)	***C ***[functional condition(true:PATH;false:PATH;value:PATH)]
Operator: Suspension/resumption (***S***)	***S ***[re-take jobs: execution]
